# Identification of Toll-Like Receptor 9 as Parapoxvirus Ovis-Sensing Receptor in Plasmacytoid Dendritic Cells

**DOI:** 10.1371/journal.pone.0106188

**Published:** 2014-08-29

**Authors:** Heiner von Buttlar, Sabine Siegemund, Mathias Büttner, Gottfried Alber

**Affiliations:** 1 Institute of Immunology, College of Veterinary Medicine, University of Leipzig, Leipzig, Germany; 2 Bavarian Health and Food Safety Authority, Oberschleissheim, Germany; Beth Israel Deaconess Medical Center, Harvard Medical School, United States of America

## Abstract

Parapoxvirus ovis (PPVO) is known for its immunostimulatory capacities and has been successfully used to generate vector vaccines effective especially in non-permissive host species. Murine conventional and plasmacytoid dendritic cells (cDC and pDC) are able to recognize PPVO. The PPVO-sensing receptor on pDC is hitherto unknown. In this study we aimed to define the pattern recognition receptor responsible for the activation of murine pDC by inactivated and replication-competent PPVO. We show that PPVO-induced expression of type I and type III interferons, pro-inflammatory cytokines, and co-stimulatory CD86 by bone marrow-derived pDC but not cDC is blocked by chloroquine, an inhibitor of endosomal maturation. The activation of pDC is independent of viral replication and depends mainly on TLR9. Moreover, the use of phosphatidylinositol 3-kinase inhibitor wortmannin or C-Jun-N-terminal kinase inhibitor SP600125 results in significant reduction of PPVO-induced pDC activation. Taken together, our data identify endosomal TLR9 as PPVO-sensing receptor in pDC.

## Introduction

Parapoxvirus ovis (PPVO), also known as orf virus, is an enveloped virus with a GC-rich genome [1], [2] and the prototype species of the genus parapoxvirus in the *Poxviridae* family. Replication-competent as well as inactivated virus preparations are known for their immunomodulating activities [3]–[6]. PPVO enhances innate immune mechanisms such as phagocytosis, the generation of reactive oxygen intermediates, pro-inflammatory cytokines and especially production of type I interferons (IFN) [4]–[7]. Recently, the induction of type III IFN in plasmacytoid dendritic cells (pDC) by PPVO has been shown [7]. Type III IFNs share the antiviral and immunomodulatory properties of type I IFNs and are known to act especially on epithelial surfaces [8], [9]. With its effects on innate immune mechanisms PPVO was shown to interfere with the replication and pathogenesis of other viruses *in vivo* and *in vitro* [10]. Moreover, with its immunomodulatory properties PPVO efficiently primes adaptive immune responses when used as a vaccine vector even in non-permissive host species [11]–[14].

Priming of the adaptive immune response requires prior activation of the innate immune response. Dendritic cells (DC) play a central role in the interaction between the innate and the adaptive immune response. Upon activation by pathogen-associated molecular patterns (PAMP), DC induce and orchestrate adaptive immune mechanisms by cytokine and co-stimulatory surface molecule expression. Conventional dendritic cells (cDC) sense PPVO independently of the TLR signalling molecules MyD88 and TRIF, whereas pDC depend on the adaptor molecule MyD88 that is used by several TLRs [6]. pDC are specialised for the detection of foreign nucleic acids by their endosomal TLRs [15]. A hallmark of the subsequent pDC activation is the production of type I and type III IFN [16]. The endosomal TLRs are transferred from the ER to the endolysosome, where they are enzymatically cleaved to gain functionality (reviewed in [17]). Mature TLR3, TLR7, and TLR8 can sense ds and ssRNA molecules, respectively, whereas TLR9 is able to interact with DNA at GC motifs [18]. Nucleic acid recognition by endosomal TLR is the underlying mechanism for IFN induction in pDC by RNA viruses (e.g. Newcastle disease virus) and DNA viruses (e.g. Herpesvirus) [19], [20]. For the *Poxviridae* two main TLR in pDC are known. While the detection of myxoma virus and ectromelia virus is TLR9-dependent [21]–[23], the highly attenuated modified vaccinia virus Ankara is sensed by DC even in absence of TLR9. Nevertheless, the stimulation of DC by UV-inactivated modified vaccinia virus Ankara relies on TLR9 [23]. Less attenuated vaccinia virus strains potently block immune stimulation. After heat inactivation these viruses activate pDC via TLR7 [21]. Thus, TLR7 and TLR9 were both potential candidates for the MyD88-dependent recognition of PPVO by pDC.

Here, after confirming the endosomal sensing of PPVO in pDC using chloroquine, we identify TLR9 as main PPVO-sensing receptor triggering the activation of pDC by using TLR9-inhibitory CpG-ODN as well as TLR9^−/−^ pDC. For PPVO-induced TLR9-dependent immune stimulation of pDC, phosphatidylinositol 3-kinase (PI3K) and C-Jun-N-terminal Kinase (JNK) signalling is shown to be necessary.

## Materials and Methods

### Viruses

Parapoxvirus ovis D1701 was propagated and titrated in bovine kidney cells and purified via sucrose gradient centrifugation. The bovine kidney cell line BK-KL 3A [24] was kindly provided by T. Schlapp, Bayer AG, Monheim, Germany. Virus batches were divided into replication-competent PPVO (PPVO) and inactivated PPVO (iPPVO), the latter was treated with beta-propiolactone for chemical inactivation. Newcastle disease virus (NDV) was propagated in embryonated egg cultures, purified by sucrose gradient centrifugation and quantified by haemagglutination assay.

### Dendritic cell culture

To generate DC *in vitro*, bone marrow (BM) of C57Bl/6 WT mice (Janvier) or TLR9^−/−^ mice (provided by Dr. S. Bauer with permission from Dr. S. Akira) on the C57Bl/6 genetic background [25] were flushed and cells were cultured for 6.5 days in RPMI with 10% fetal bovine serum, 1% sodium pyruvate, 50 µM 2-mercaptoethanol and 50 ng/ml fms-like tyrosine kinase 3 ligand (Flt3L) to reach high numbers of pDC [26]. Cells were stained and purified using a FACSAriaIII into cDC (CD11c^+^; CD11b^+^; B220^−^) and pDC (CD11c^+^; CD11b^+/−^; B220^+^) to a purity of at least 98%. Pure subpopulations were stimulated for 24 h with the indicated stimuli. Supernatants were harvested for determination of cytokine levels by ELISA and cells were stained and analysed for activation marker expression on a BD Fortessa.

### Ethics statement

All bone marrow samples were derived from euthanized mice. Mice were euthanized with CO_2_. The protocol for this study was approved by the Animal Care and Usage Committee of the Landesdirektion (state office) Sachsen in Leipzig, Germany (Permit number: T 01/14). All efforts were made to minimize animal suffering.

### Stimuli and inhibitors

Cells were stimulated with sucrose gradient-purified PPVO that was left untreated or chemically inactivated by beta-propiolactone at a MOI of 10. Purified Newcastle disease virus (NDV), CpG-ODN 2216 (5-GsGsGGGACGATCGTCsGsGsGsGsGsG-3; s = phosphothioate) (1 µM; Enzo Life Sciences, Lausen, Switzerland) and medium stimulation served as controls. For the competitive inhibition of TLR9, G-type inhibitory ODN (sequence 5′-ctcctattggggtttcctat-3′) (5 µM; Enzo Life Sciences, Lausen, Switzerland) were added to the stimulations. Endosomal maturation was prevented by the addition of chloroquine (0.4 µg/ml; Sigma Aldrich, St. Louis, MO, USA). To interfere with PI3K and JNK signalling, chemical inhibitors wortmannin (1 µM; Sigma Aldrich, St. Louis, MO, USA) and SP600125 (up to 10 µM; Sigma-Aldrich, St. Louis, MO, USA) were used.

### Antibodies and ELISA

For surface marker staining the following antibodies were used: anti-mouse CD11c (clone N418); anti-mouse CD11b (clone M1/70); anti-mouse B220 (clone RA3-6B2); anti-mouse CD86 (clone GL1); anti-mouse MHC-II I-A/I-E (clone M5/114.15.2) and respective isotype controls (all: ebioscience, Frankfurt, Germany). IFNs and cytokines were detected in sandwich-ELISA with the following reagents: IFN-α: anti-mouse IFN-α antibody (clone: RMMA-1), polyclonal rabbit anti-mouse IFN-α-purified immunoglobulin G (R&D Systems GmbH, Wiesbaden, Germany); IFN-β: anti-mouse IFN-β antibody (clone: RMMB-1), polyclonal rabbit anti-mouse IFN-α-purified immunoglobulin G (R&D Systems GmbH, Wiesbaden, Germany); IL-12p40: anti-mouse IL-12p40 antibody (clone: 5C3), biotinylated goat anti-mouse IL-12p40-purified IgG (provided by M. Gately, F. Hoffmann-La Roche Ltd., Nutley, NJ); IL-12p40 ELISA standard was kindly provided by Schering-Plough Biopharma (Palo Alto, CA, USA); IL-6: anti-mouse IL-6 antibody (clone MP5-20F3; BD Biosciences, Heidelberg, Germany), biotinylated anti-mouse IL-6 antibody (clone: MP5-32C11; BD Biosciences, Heidelberg, Germany); Interferon λ: Mouse IL-28A/B (IFN-lambda 2/3) DuoSet (R&D Systems GmbH, Wiesbaden, Germany).

### Statistics

For the determination of statistically significant differences 2-way ANOVA and Bonferroni post-test were done with GraphPadPrism 5. Differences are marked by asterisks (*p<0.05; **p<0.01; ***p<0.001).

## Results

### Endosomal maturation is a prerequisite for PPVO-induced activation of pDC, but not of cDC

**Figure 1:**
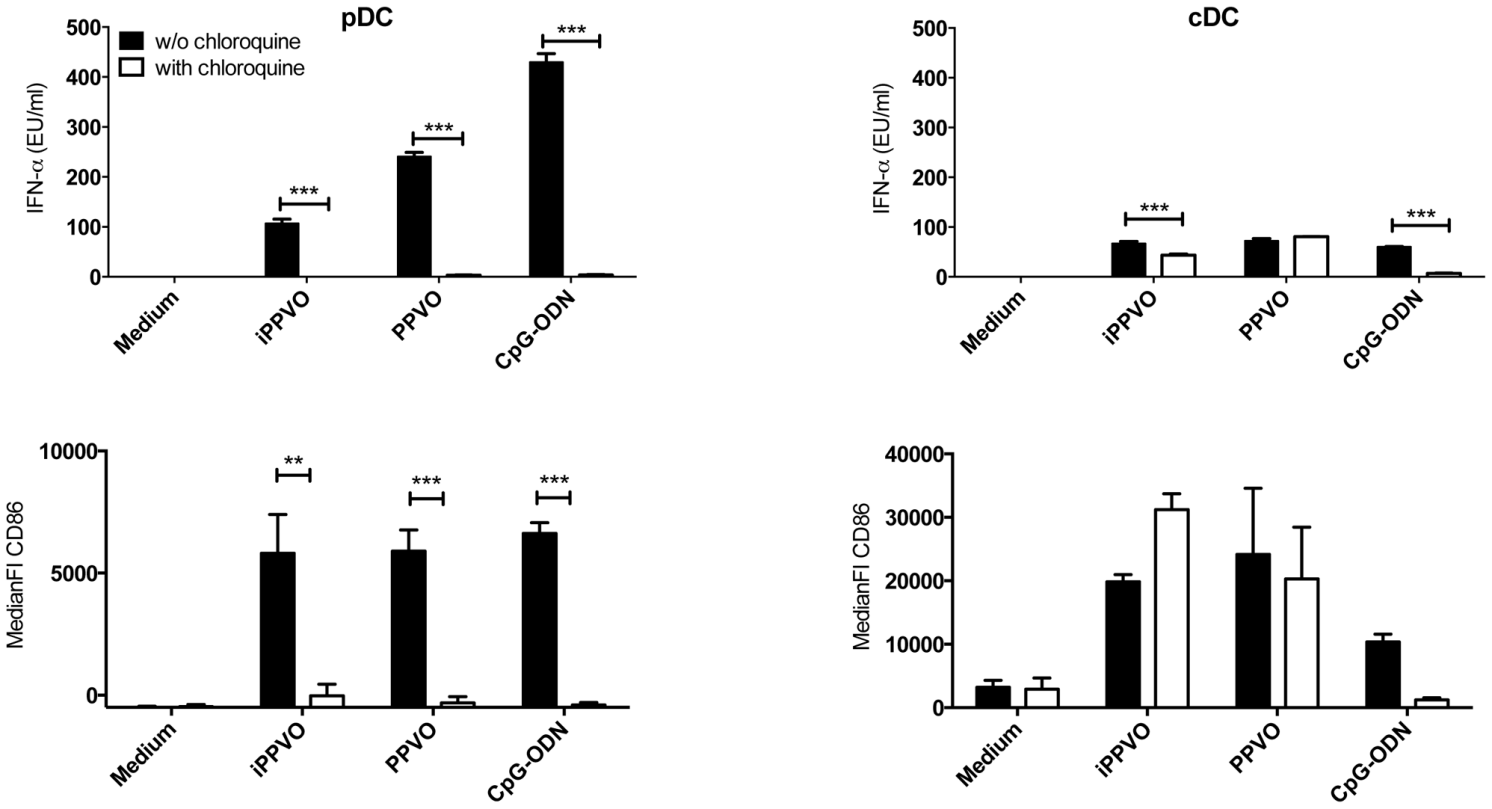
PPVO sensing requires endosomal maturation in pDC but not in cDC. BMDC were generated in the presence of Flt3-ligand and pDC and cDC purified by FACS as described in material and methods. Purified pDC and cDC were stimulated in triplicates as indicated for 24 h in the absence or presence of chloroquine and supernatants were analysed for the indicated cytokines. Concentrations of cytokines are presented as mean +/− SEM. One representative experiment of three is presented for cytokine data. Remaining cells were stained and analysed for the expression of co-stimulatory CD86 presented as mean +/− SEM of the median fluorescence intensity. Pooled data of two experiments are shown for CD86 expression. Statistically significant differences between stimulations in the absence and presence of chloroquine were determined by 2-way ANOVA and Bonferroni post-test and are indicated by asterisks.

In a previous study we showed that chemically inactivated PPVO (iPPVO) activates conventional dendritic cells (cDC) in a MyD88- and TRIF-independent manner, whereas the activation of plasmacytoid dendritic cells (pDC) relies on TLR-related signal adaptor MyD88 [6]. pDC especially express endosomal TLRs. A prerequisite for endosomal TLR7, 8 and 9 function is their cleavage after endosome maturation [27]. We tested whether endosomal maturation is necessary for PPVO-mediated pDC and cDC activation. Highly purified (>98%) BM-pDC (CD11c^+^CD11b^+/−^B220^+^) and BM-cDC (CD11c^+^CD11b^+^B220^−^) generated from bone marrow cells in the presence of fms-like tyrosine kinase 3 ligand (Flt3L) were stimulated with replication-competent PPVO, iPPVO and the control stimulus CpG-ODN in the absence and presence of chloroquine, an inhibitor of endosome maturation. As expected, activation of BM-pDC and BM-cDC by TLR9-dependent control stimulus CpG-ODN was abolished in the presence of chloroquine. In BM-pDC the blockade of endosome maturation also largely diminished upregulation of co-stimulatory CD86 and abolished the secretion of IFN-αβ, IFN-λ and pro-inflammatory cytokines to both PPVO preparations. In contrast to pDC, purified BM-cDC were activated by replication-competent PPVO and iPPVO, reflected by cytokine secretion and upregulation of surface markers CD86 and MHC-II, even in the presence of chloroquine (Figure 1 and data not shown). Thus, whereas PPVO is sensed in BM-cDC outside of the endosomal compartment, BM-pDC recognize PPVO with an endosomal receptor.

### TLR9-inhibitory iCpG-ODN antagonize PPVO-induced IFN-α and IFN-λ production by purified plasmacytoid dendritic cells

**Figure 2:**
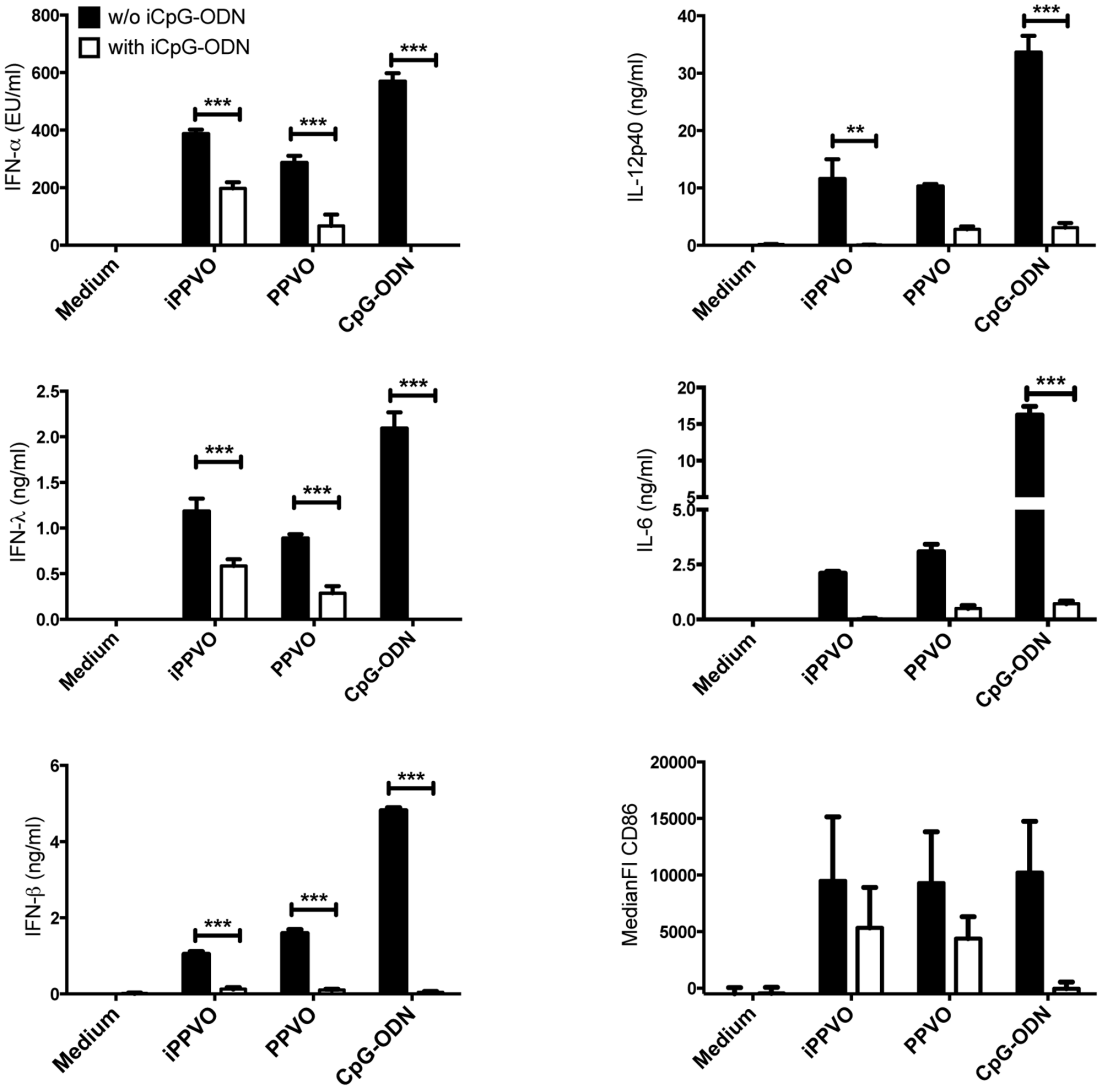
PPVO-induced pDC activation is blocked by TLR9-specific iCpG-ODN. Purified BM-pDC were stimulated in triplicates as indicated for 24 h in the absence or presence of iCpG-ODN (iCpG) and supernatants were analysed for the indicated cytokines. Concentrations of cytokines are presented as mean +/− SEM. One representative experiment of three is presented for cytokine data. Remaining cells were stained and analysed for the expression of co-stimulatory CD86 presented as the mean +/− SEM of the median fluorescence intensity. Pooled data of three experiments are shown for CD86 expression. Statistically significant differences between stimulations in the absence and presence of iCpG-ODN (5 µM) were determined by 2-way ANOVA and Bonferroni post-test and are indicated by asterisks.

Taking the high GC content [1], [2] of the parapoxviral genome into account, the endosomal DNA-recognizing receptor TLR9 might be involved in sensing PPVO in pDC. To test this hypothesis, we stimulated purified BM-pDC in the absence or presence of TLR9-specific inhibitory (i)CpG-ODN. Replication-competent PPVO and iPPVO induced considerable levels of IFN-α and IFN-λ in BM-pDC. The specific blockade of TLR9 significantly reduced secretion of IFN-α and IFN-λ by BM-pDC in response to replication-competent PPVO, iPPVO, and CpG-ODN (Figure 2). Additionally, the secretion of IL-12p40 or IL-6 by pDC was induced by both, replication-competent and inactivated PPVO preparations as well as CpG-ODN. The presence of TRL9-specific iCpG-ODN led to lower levels of these pro-inflammatory cytokines (Figure 2).

Upon activation, DC present antigen via MHC molecules and express co-stimulatory surface antigens such as CD86 and thereby activate adaptive immune mechanisms. PPVO is able to induce the upregulation of MHC-II and CD86 expression on BM-pDC [6]. TLR9-specific iCpG-ODN reduced the ability of BM-pDC to up-regulate CD86 (Figure 2) and MHC-II (data not shown) surface expression in response not only to CpG-ODN but also to replication-competent PPVO and iPPVO.

Taken together, these data provide evidence for a substantial involvement of TLR9 in PPVO recognition and subsequent pDC activation.

### Reduced PPVO-induced IFN-α and IFN-λ production by purified TLR9^−/−^ plasmacytoid dendritic cells

**Figure 3:**
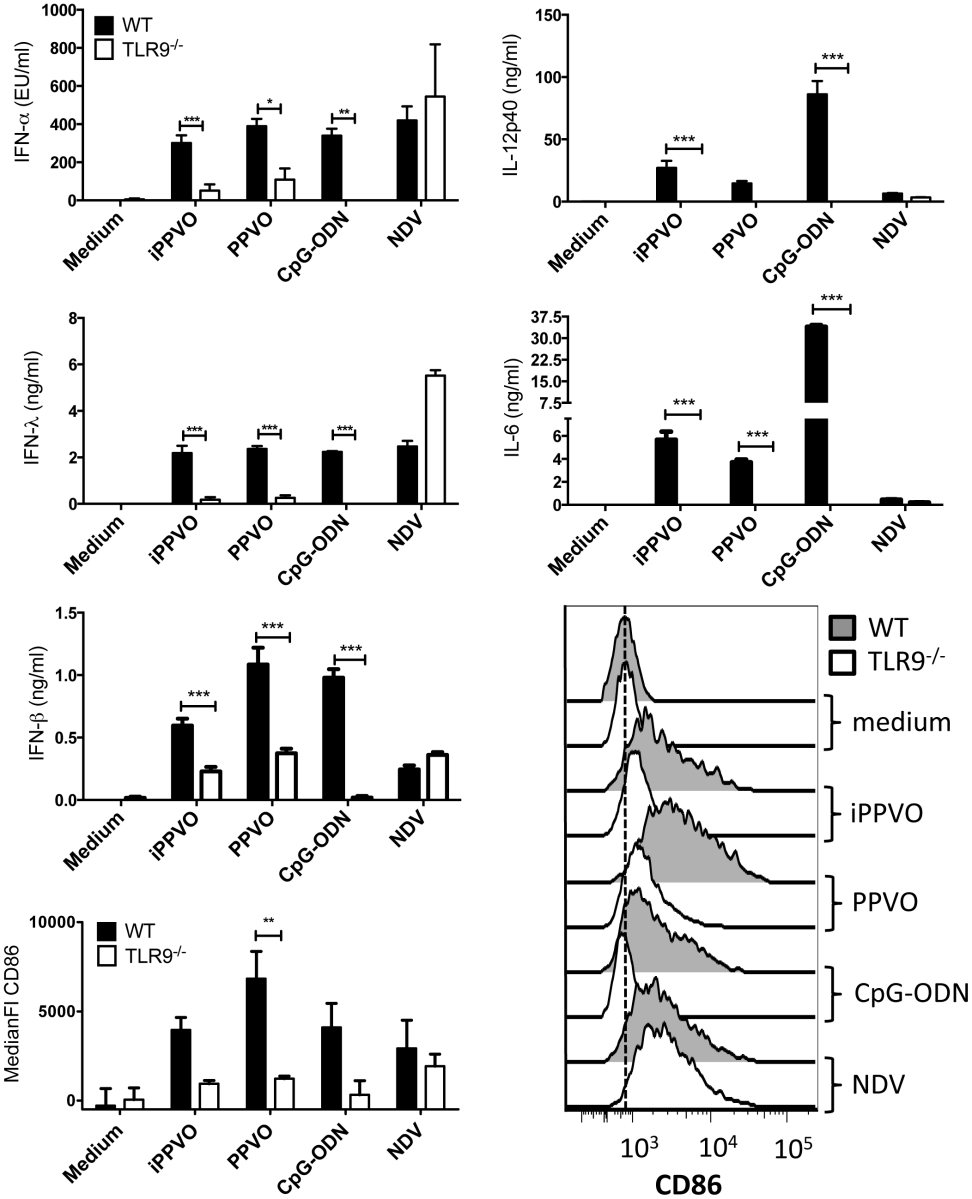
Stimulation of pDC by PPVO is greatly diminished in the absence of TLR9. BMDC of WT and TLR9^−/−^ mice were generated and purified as described in material and methods. Purified pDC were stimulated in triplicates with the indicated stimuli and supernatants and cells were harvested after 24 h. Cytokines were determined in culture supernatants using ELISA and expression of CD86 by flow cytometry. One representative of three independent experiments is depicted for cytokine data. Histograms of one representative and pooled data of three independent experiments are shown for CD86 expression. Cytokine concentrations and CD86 expression were statistically analyzed using 2-way ANOVA and Bonferroni post-test, and significant differences between WT and TLR9^−/−^ pDC responses are indicated by asterisks.

Competitive blocking of TLR9 by iCpG-ODN resulted in substantially reduced but not completely abrogated PPVO-mediated pDC activation. This suggests either an incomplete inhibition of TLR9 by iCpG-ODN or a redundant receptor capable of sensing PPVO. To distinguish these two possibilities, we used BMDC from TLR9^−/−^ mice [25]. BM-pDC from TLR9^−/−^ mice were incubated with replication-competent and inactivated PPVO preparations as well as CpG-ODN. TLR9^−/−^ BM-pDC secreted significantly less IFN-α and IFN-λ than wild type (WT) BM-pDC. IFN-β levels were not reduced as prominently in response to replication-competent and iPPVO (Figure 3). It is noteworthy that especially after stimulation with replication-competent PPVO, TLR9^−/−^ BM-pDC still are able to produce residual IFN-α and IFN-λ amounts. This confirms our previous result with TLR9-inhibiting iCpG-ODN leading to a substantial but not complete reduction of IFN production.

TLR9^−/−^ BM-pDC produced also less of the pro-inflammatory cytokines IL-12p40 and IL-6 and up-regulated CD86 surface expression to a lower degree than WT BM-pDC upon encounter with replication-competent PPVO, iPPVO or CpG-ODN, whereas TLR7-dependent NDV [20] activated both TLR9^−/−^ and WT BM-pDC (Figure 3). This and the IFN-β production in response to PPVO by TLR9^−/−^ BM-pDC demonstrate that TLR9^−/−^ BM-pDC are capable to produce WT levels of IFNs. Thus, the reduced secretion of IFN-α, IFN-λ, pro-inflammatory IL-12p40 and IL-6 as well as MHC-II and CD86 expression in response to PPVO is TLR9-specific. Even though the residual activation of TLR9^−/−^ pDC indicates the presence of additional receptors sensing PPVO, these data clearly identify TLR9 as the predominant PPVO-recognizing receptor in pDC.

### Inhibition of PI3K and JNK signalling leads to reduced activation of pDC by PPVO

**Figure 4:**
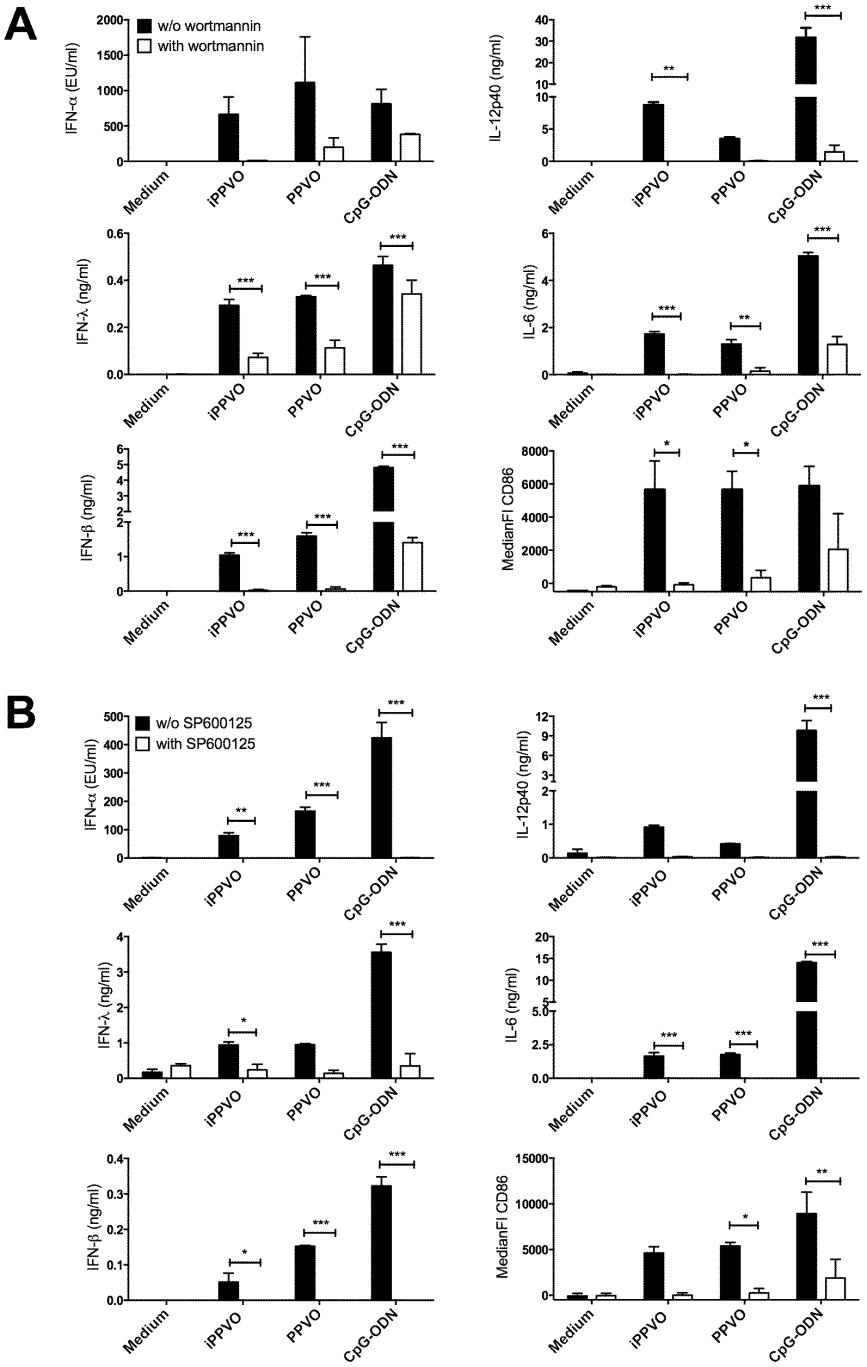
Inhibition of PI3K and JNK signalling greatly diminishes activation of pDC by PPVO. BMDC of WT mice were generated and purified as described in material and methods. Purified pDC were stimulated in triplicates with the indicated stimuli in the absence or presence of PI3K inhibitor wortmannin (A) or JNK inhibitor SP600125 (10 µM) (B) and supernatants and cells were harvested after 24 h. Cytokine concentrations were determined in culture supernatants using ELISA and are depicted as mean +/− SEM. Surface expression of CD86 was analysed by flow cytometry and is depicted as mean +/− SEM of the median fluorescence intensity. Cytokine concentrations and CD86 expression were statistically analysed using 2-way ANOVA and Bonferroni post-test and differences are indicated by asterisks. One representative experiment of at least three is presented for cytokine data. Pooled data of two (A) or three (B) experiments are shown for CD86 expression.

Phosphatidylinositol-3 kinases (PI3Ks) are required for IFN-α induction by CpG-ODN in human pDC, but do not regulate pro-inflammatory cytokine production or costimulatory molecule expression [28]. The stimulation with PPVO in the presence of the PI3K inhibitor wortmannin led not only to reduced IFN-α and IFN-λ secretion but also diminished IL-12p40 and IL-6 secretion as well as CD86 expression by pDC (Figure 4A). Moreover we interfered with JNK signalling, known to be involved in TLR-mediated induction of pro-inflammatory cytokines in DC [29], by using the JNK inhibitor SP600125. As shown in Figure 4B, this was accompanied by reduction of the secretion of type I and type III IFNs upon PPVO, iPPVO and to a lesser extent upon CpG-ODN stimulation. Production of pro-inflammatory cytokines IL-6 and IL-12p40 as well as upregulation of costimulatory CD86 was also affected (Figure 4B). Thus, our data suggest that activation of pDC by PPVO involves PI3K and JNK signalling.

## Discussion

Parapoxvirus ovis (PPVO), replication-competent and inactivated, is a potent stimulator of innate immune responses in permissive and non-permissive species as well as a viral vector shown to effectively induce adaptive immune responses [3], [11], [12], [30]. A hallmark of the immune stimulation is the induction of high amounts of type I interferons (IFN) [3]. IFN as well as pro-inflammatory cytokines are secreted by pDC as well as cDC. For the recognition of DNA viruses like herpes simplex virus (HSV)-1 or adenovirus, TLR-dependent and TLR-independent pathways are engaged [19], [31]. Also for PPVO-mediated activation of cDC, TLR-dependent and -independent mechanisms were shown to be important [6]. However, the receptors sensing PPVO remained elusive. Our data show that chloroquine, an inhibitor of endosomal maturation, prevents PPVO-induced activation of pDC but not cDC (Figure 1). This is consistent with our previous observation of MyD88-independent cDC activation [6] and points to endosomal pDC activation by PPVO (Figure 1).

pDC are specialised to sense nucleic acids [15]. *Poxviridae* such as ectromelia virus and myxoma virus are sensed by the DNA-recognising TLR9 [21], [23]. However, DNA viruses such as HSV-1 or heat-inactivated vaccinia virus are also sensed by pDC in a TLR9-independent fashion [19], [21]. The induction of IFN by vaccinia virus requires the RNA sensor TLR7 [21]. Similarly, the activation of BM-DC by the highly attenuated strain modified vaccinia virus Ankara is mainly independent of TLR-related signalling molecules MyD88 and TRIF [32]. Vaccinia virus subverts the action of cytoplasmic nucleic acid sensors such as protein kinase R by sequestering nucleic acids by its Z-DNA/RNA binding protein E3 [21]. This protein is also able to counteract the detection of myxoma virus and CpG-ODN by TLR9 [21]. Parapoxvirus ovis encodes for a homologue of vaccinia virus E3, i.e. OV20.0L, that in contrast to myxoma virus homologue M029L shares the Z-DNA binding domain of vaccinia virus E3 [33]–[35]. Thus, to successfully sense PPVO, DC might employ multiple receptors and signalling pathways.

We demonstrate the dependence of PPVO, with its genome rich in GC motifs [1], [2], on TLR9 for the activation of pDC by using iCpG-ODN and TLR9^−/−^ cells (Figures 2 and 3). Thus, in contrast to TLR7-dependent sensing of heat-inactivated vaccinia virus, pDC mainly rely on TLR9 for the recognition of PPVO similarly as it was shown for myxoma virus. [21]. In contrast to HSV-1, another DNA virus, the activation of pDC in response not only to inactivated virus but also to replication-competent PPVO mainly relies on TLR9 in the non-permissive murine pDC [14]. We therefore believe that the PPVO DNA is the viral component activating pDC. Potential early gene expression by inactivated PPVO does not seem to play a major role for the activation of pDC. Studies reported profoundly reduced early gene transcriptional activity following virus inactivation [24], [36], [37], however, we observed no major differences between inactivated and replication-competent PPVO preparations in pDC activation.

PI3K inhibitor wortmannin prevents the TLR9-dependent activation of pDC by PPVO (Figure 4A). PI3K signalling has been shown to be a requirement for nuclear translocation of IRF7 in response to CpG-ODN, thus facilitating type I IFN production by human and murine pDC and also plays a role for HSV-, influenza- and myxoma virus-induced IFN-α induction in pDC [21], [28]. However, there is only limited data on the role of PI3K signalling in virus infections. We show that PI3K signalling is a requirement for the immune stimulation of pDC by PPVO. Moreover, we found a reduction of all analysed activation parameters induced by PPVO after inhibiting JNK signalling with SP600125 (Figure 4B). The JNK signalling cascade is known to be involved in the induction of pro-inflammatory cytokines by TLR [29], but the JNK inhibitor SP600125 only marginally influences CpG-ODN-induced matrixmetalloproteinase 13 production by odontoblasts or RANTES secretion by Langerhans cells [38], [39]. We demonstrate that SP600125 inhibits PPVO-mediated type I IFN induction by TLR9 in pDC. However, the data shown here leave open whether wortmannin and SP600125 inhibit type I IFN induction by TLR9 in pDC directly or indirectly.

Besides their action on TLR signalling, both wortmannin and SP600125 are known to interfere with autophagosome formation. PPVO like other poxviruses replicates in the cytoplasm. Vaccinia virions are known to deliver their core via the plasma membrane or endosomal membrane into the cytoplasm where the genomic DNA is liberated [40], [41]. Thus, poxviral genomic DNA is unlikely to be accessible for TLR9 after endosomal uptake of the virion. For the interaction of TLR9 with parapoxviral DNA, its uptake from the cytosol into autophagosomes could be a possible mode of action. Whether poxviruses with their cytoplasmic replication cycle share autophagic sampling of their nucleic acids for endosomal TLR-dependent sensing with RNA viruses e.g. respiratory syncytial virus, vesicular stomatitis virus or paramyxovirus, needs further investigation [38], [42], [43].

In summary, our data identify endosomal TLR9 as the main receptor recognising PPVO in pDC. For characterization of the TLR-independent activation of cDC by PPVO [6], identification of the non-TLR pattern recognitions receptors expressed by cDC and responsible for PPVO-mediated cDC activation merits further investigation. This will help to comprehensively understand the various immunomodulatory properties of PPVO making it an efficient viral vaccine vector.
